# The potential of the antifungal nystatin to be repurposed to fight the protozoan *Trypanosoma cruzi*

**DOI:** 10.3389/fmicb.2025.1539629

**Published:** 2025-03-12

**Authors:** Belén Jesús Maciel, Chantal Reigada, Fabio Augusto Digirolamo, Marcos Rengifo, Claudio Alejandro Pereira, Mariana Reneé Miranda, Melisa Sayé

**Affiliations:** ^1^Universidad de Buenos Aires, Facultad de Medicina, Instituto de Investigaciones Médicas A. Lanari, Buenos Aires, Argentina; ^2^Consejo Nacional de Investigaciones Científicas y Técnicas, Universidad de Buenos Aires, Instituto de Investigaciones Médicas (IDIM), Laboratorio de Parasitología Molecular, Buenos Aires, Argentina

**Keywords:** nystatin, drug repurposing, *Trypanosoma cruzi*, trypanocidal compounds, Chagas disease

## Abstract

Chagas disease, caused by the parasite *Trypanosoma cruzi*, affects 6 million people worldwide. Although the drugs benznidazole (BZN) and nifurtimox are available to treat Chagas, they are not effective in the chronic phase when most patients are diagnosed. Moreover, long-term regimen and severe side effects often lead to poor adherence and treatment abandonment. These problems highlight the urgent need to develop new therapies to treat this neglected disease. Given that the antifungal drug nystatin (NYS) affects arginine uptake in yeasts, and fluctuations on arginine availability through transport processes in *T. cruzi* can negatively affect its viability, in this work we evaluated the potential of NYS for drug repurposing against *T. cruzi*. NYS inhibited arginine uptake and presented trypanocidal effect in both epimastigotes (IC50 0.17 μM) and trypomastigotes (IC50 4.90 μM). In addition, treatment of infected cells with NYS decreased the release of trypomastigotes with better efficacy than BZN (IC50s 4.83 μM and 8.60 μM, respectively) suggesting that NYS affects the progression of the intracellular life cycle. Furthermore, we observed a synergistic effect both in isolated trypomastigotes and infected cells when NYS was combined with BZN, which could enhance efficacy while improving treatment safety and adherence. As in yeasts, the mechanism of action of NYS in *T. cruzi* involved the plasma membrane disruption, and membrane transport processes, like amino acids and thymidine uptake, were affected prior to the disruption probably due to NYS interaction with the membrane. Drug repurposing is a recommended strategy by the World Health Organization to develop new therapeutic alternatives for neglected diseases. Our results indicate that NYS presents great potential to be repurposed as a trypanocidal drug to fight *T. cruzi*.

## 1 Introduction

Chagas disease, caused by *Trypanosoma cruzi*, affects 6 million people worldwide and other 70 million are at risk of getting infected (World Health Organization, 2015). Only two medications are available to treat this neglected disease, nifurtimox and benznidazole (BZN). Both were discovered more than half century ago and the long-term therapies are associated with severe side effects that often lead to treatment abandonment. Moreover, these drugs are not effective in the chronic stage when most patients are diagnosed (Rassi et al., 2010; Zuma and de Souza, 2021) and the randomized trial BENEFIT (BENznidazole Evaluation For Interrupting Trypanosomiasis) further demonstrated that BZN does not halt disease progression (Morillo et al., 2015). These problems highlight the urgent need to develop new alternative therapies to treat Chagas disease.

Nystatin (NYS) is a polyene drug with antifungal and fungistatic activity that is used to treat several yeasts and fungi infections in humans. In addition to sterol binding and pore formation (Kristanc et al., 2019), NYS inhibits arginine uptake by targeting the arginine specific transporter CAN1 in *Saccharomyces cerevisiae* (Stachiewicz and Quastel, 1963; Opekarová and Tannerb, 1994). The structure, chemistry, action mechanism, and resistance of NYS resemble those of amphotericin B, a drug used to treat leishmaniasis, another neglected disease caused by the trypanosomatid *Leishmania* spp. (Kumari et al., 2022). NYS is included in the 23rd Model List of Essential Medicines (2023) as a safe and cost-effective medicine for the healthcare system (World Health Organization, 2023). Drug repurposing, which refers to the identification of new clinical applications for approved or investigational drugs, is a strategy recommended by the World Health Organization to accelerate the development of treatments for neglected diseases. This approach reduces time and investment costs because safety profile and pharmacological properties, for example, are already known (Ashburn and Thor, 2004).

Amino acid metabolism plays a crucial role in the parasite *T. cruzi*, since amino acids are involved in a wide variety of essential processes like protein synthesis, osmoregulation, stress resistance and parasite stage differentiation, among others (Silber et al., 2005). Particularly, arginine plays a unique role in *T. cruzi* as it participates in the cell energy management through arginine kinase and phosphoarginine (Pereira et al., 2000). Arginine can be obtained through protein degradation or active uptake and *T. cruzi*’s viability can be affected through fluctuations in arginine availability (Miranda et al., 2012). Furthermore, RNAi assays in *T. brucei* have demonstrate the essentiality of its arginine permease TbAAT5-3 (Mathieu et al., 2017).

Given the relevance of arginine in *T. cruzi*, its metabolism and availability can be considered promising therapeutic targets. A bibliographical search for compounds that inhibit arginine uptake or affect its intracellular concentration in different organisms allowed the identification of NYS. In this work, NYS was evaluated as an arginine transport inhibitor and as trypanocidal compound in *T. cruzi* in order to investigate its potential for drug repurposing against this parasite.

## 2 Methods

### 2.1 Parasites

Epimastigotes of CL Brener strain were cultured at 28°C in plastic flasks containing brain-heart infusion-tryptose (BHT) medium supplemented with 10% fetal calf serum (FCS), 100 U/mL penicillin, 100 μg/mL streptomycin and 20 μg/mL hemin (Camargo, 1964).

### 2.2 Mammalian cell lines

HEK293 (human embryo kidney, ATCC CRL-1573), 3T3-L1 (mouse embryo fibroblast, ATCC CL-173) and Vero cells (african green monkey kidney, ATCC CCL-81) were cultured in Minimal Essential Media (MEM) supplemented with 10% FCS, 0.15% (w/v) NaHCO_3_, 100 U/mL penicillin and 100 μg/mL streptomycin at 37°C, 5% CO_2_. Vero cells (5 × 10^4^ per well) were infected with trypomastigotes (2.5 × 10^5^ per well) for 24 h. After this period, the infected cells were washed twice with phosphate buffered saline (PBS), the MEM medium was replaced for MEM 4% FCS, and the cells were kept in culture at 37°C, 5% CO_2_. Trypomastigotes were obtained from infected Vero cells as previously described (Tonelli et al., 2004).

### 2.3 Drugs

NYS was purchased from Magel (Magel SA Import Medicinal Drugs, Argentina) and benznidazole (BZN) was purified from ABARAX^®^ (Elea Laboratorios, Argentina). Stock solutions were made in DMSO (NYS, 50 mM; BZN, 100 mM) and dilutions were prepared in the same solvent. DMSO did not exceed 1% final concentration, and was also used as no drug control in experiments.

### 2.4 Metabolites transport assay

1 × 10^7^ epimastigotes were centrifuged at 8,000 × g for 30 s, and washed once with PBS. Cells were resuspended in 0.1 mL PBS and the assay started by the addition of 0.1 mL of the transport mix containing L-[^3^H] arginine, [^3^H] thymidine or [^3^H] amino acid mix (aspartate, glutamate, glutamine, glycine, leucine, lysine, phenylalanine, serine, tryptophan and valine) (PerkinElmer’s NEN Radiochemicals; 0.4 μCi) (Sayé et al., 2017). Following 15 min incubation at 28°C, reaction was stopped by adding 1 mL of ice-cold PBS. Cells were centrifuged as indicated above, and washed twice. Cell pellets were resuspended in 0.2 mL of water and counted for radioactivity in UltimaGold XR liquid scintillation cocktail (Packard Instrument Co., Meridien CT, United States). Non-specific uptake and carry over were assayed without incubation (T0), or incubated at 4°C. Normal parasite motility and morphology were confirmed by direct microscopic examination after incubation with the drug.

### 2.5 Trypanocidal activity

1 × 10^7^ epimastigotes/mL in exponential growth phase were cultured in BHT medium with increasing concentrations of NYS and BZN in a 96-well plate to a final volume of 200 μL at 28°C. Parasite growth was determined after 24 h by counting in Neubauer chamber (Galceran et al., 2023).

1 × 10^6^ trypomastigotes/mL were incubated with increasing concentrations of the drugs in a 96-well plate to a final volume of 200 μL for 24 h at 37°C. Survival was determined by counting in a Neubauer chamber. Only swimming parasites were counted (Galceran et al., 2023).

### 2.6 Membrane integrity evaluation

#### 2.6.1 Membrane permeabilization

Membrane permeabilization assay was performed as previously described (Reigada et al., 2017). Briefly, 10^8^ epimastigotes were incubated in PBS with increasing concentrations of NYS (0, 0.75, 1.0, 1.25, 1.5 and 2.0 μM). After 30 min of incubation with the drug at room temperature, the tubes were centrifuged, supernatants were kept on ice and pellets were resuspended in the same buffer. Permeabilization with digitonin was used as a positive control; cells were washed twice and resuspended in 50 mM Tris-HCl buffer, pH 7.5, 0.25 M sucrose and 10 μM E64. 10^8^ parasites were mixed with 100 μL of the same buffer containing 0.3 mg/mL of digitonin. After incubation at room temperature, the tubes were centrifuged, supernatants were transferred to new tubes and pellets were resuspended in the same buffer. Samples of all supernatant and pellet fractions corresponding to 0.5 × 10^7^ parasites were run on 15% SDS-PAGE gels and transferred onto a PVDF membrane. Western blot was performed with primary rabbit antibodies anti-glutamate dehydrogenase (1:5,000 dilution) followed by incubation with peroxidase-conjugated anti-rabbit (1:5,000 dilution). The peroxidase-labeled proteins were revealed using Super Signal West Pico Chemiluminescent substrate following the manufacturer instructions (Pierce, Waltham, MA, United States).

#### 2.6.2 Membrane integrity

After treatment with NYS, membrane integrity was determined by ethidium bromide (EtBr) exclusion as described by Schoijet et al. (2008). Briefly, 10^7^ epimastigotes resuspended in PBS were incubated with 50 μM EtBr for 5 min. Then, increasing concentrations of NYS (0, 0.5, 0.75, 1.0, 1.25, 1.5, 2.0, 2.5 and 3.0 μM) were added and fluorescence was monitored for 10 min. Finally, Triton X-100 0.1% was used to achieve maximum fluorescence (total loss of membrane integrity) and once it was added to the samples, fluorescence was monitored for another 10 min. EtBr fluorescence was recorded (λ_excitation_ 365 nm, λ_emission_ 580 nm) with a Sinergy HTX (Biotek) spectrofluorimeter. Linear regression was applied for each concentration and the slopes were compared to the control condition (NYS 0 μM).

### 2.7 Cytotoxicity

The toxicity of NYS was determined in HEK293, 3T3-L1 and Vero cells by the gentian violet staining assay as previously described (Reigada et al., 2019). Briefly, 1 × 10^4^ cells/well were loaded onto a 96-well plate with MEM FCS 10% and maintained for 24 h at 37°C. Medium was replaced with 150 μL MEM containing increasing concentration of the drug and incubated for other 24 h. Cells were fixed and stained with 0.5% gentian violet. Cell viability was measured by absorbance at 570 nm. The selectivity index (SI) was calculated as the ratio between the IC50 obtained in mammalian cells and the IC50 for trypomastigotes.

### 2.8 Trypomastigote release

3 × 10^4^ Vero cells/well were settled on a 24-well plate with MEM 10% FCS 500 μL, and cells were infected with 3 × 10^5^ trypomastigotes/well for 24 h. Then, medium was replaced with 500 μL of MEM 4% FCS containing increasing concentrations of NYS and BZN or their combinations and after 24 h of incubation the drugs were removed. Trypomastigotes were counted at days 8 and 9 post infection in a Neubauer chamber (Galceran et al., 2023). From day 6 post infection medium containing trypomastigotes was totally removed from the wells and then replaced with fresh MEM. Day 9 post infection was selected to report the IC50 values.

### 2.9 Combined treatment

Trypomastigotes were treated with NYS and BZN following the same procedure described in 2.5. The concentrations were chosen below the IC50 values of each drug alone and combined in a 3 × 3 matrix. BZN: 0.1 μM, 0.5 μM and 1.0 μM; NYS: 0.1 μM, 0.5 μΜ and 1.0 μM. The two best combinations were also evaluated on infected Vero cells to study the effect on the trypomastigote release according to the procedure described in 2.8. Calculations were performed using CompuSyn software^1^ which is based on the median-effect equation (Chou, 1976) and its extension, the combination index (CI) recognized as the standard measure of combination effect (Chou and Talalay, 1984). Results are reported as CI, where CI < 1, CI = 1 and CI > 1 indicate synergism, additive effect and antagonism, respectively. The fraction affected (Fa) represents the fraction of parasites affected by the drugs (0–1), where 0 indicates no effect and 1 total effect (100% death). Dose-reduction index (DRI) represents the folds a dose of each drug in a synergistic combination may be reduced when compared with the dose of each drug alone to achieve the same effect. DRI >1 indicates favorable dose-reduction.

### 2.10 Statistical analysis

IC50 values were obtained by non-linear regression of dose-response logistic functions using GraphPad Prism 6.01 for Windows. Results are presented as IC50 value with their corresponding 95% confidence interval (95% CI). Slopes for EtBr exclusion were obtained by linear regression also using GraphPad Prism 6.01 for Windows. Combined treatment was analyzed using the free software CompuSyn as mentioned in section 2.9. The parameters CI and DRI are expressed as ± standard deviation (SD). All experiments were performed at least in triplicate.

## 3 Results

### 3.1 NYS effect on *Trypanosoma cruzi* epimastigotes

**Figure 1:**
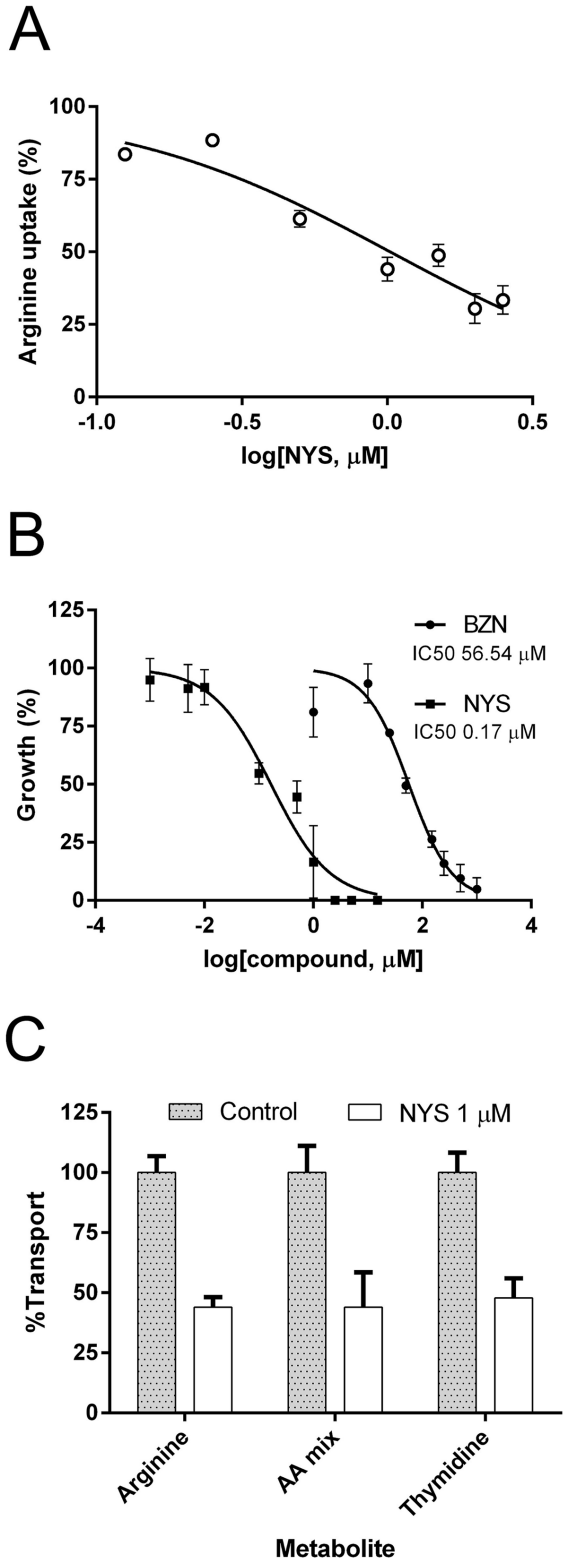
Effect of NYS on *T. cruzi* epimastigotes. **(A)** Inhibition of arginine uptake was evaluated using increasing concentration of NYS (from 0 to 2.5 μM). **(B)** Trypanocidal activity was evaluated incubating epimastigotes with concentrations of NYS (■, 0–15 μM) or BZN (●, 0–1,000 μM) for 24 h. Symbols represent mean values and error bars correspond to standard deviations (*n* = 3). Concentrations of drug inhibiting the 50% of arginine uptake and parasite growth (IC50) were calculated using the non-linear regression of dose-response logistic functions with GraphPad Prism software. **(C)** Uptake of different metabolites was evaluated in presence of 1 μM NYS. The radiolabeled amino acid mixture contained Asp, Glu, Gln, Gly, Leu, Lys, Phe, Ser, Trp, and Val. Graph shows mean values ± standard deviations (*n* = 3). NYS, nystatin; BZN, benznidazole; AA, amino acid.

Since NYS was identified through a bibliographical search of arginine uptake inhibitors, we first studied its effect on arginine transport in *T. cruzi*. By using increasing amounts of the drug, the concentration of NYS that inhibits 50% (IC50) of the transport activity in epimastigotes was calculated to be 1.02 μM (95% CI: 0.87–1.21) (Figure 1A).

The potential trypanocidal action of NYS was also assayed, yielding an IC50 of 0.17 μM (95% CI: 0.11–0.25) for epimastigote growth at 24 h (Figure 1B). The drug BZN was used as reference for trypanocidal effect as it is the current treatment for Chagas disease. Under the same experimental conditions, the IC50 for BZN was higher than the obtained for NYS with a value of 56.54 μM (95% CI: 45.05–70.94) (*p*-value <0.0001).

The large difference observed between the IC50 values for transport and growth suggests that trypanocidal effect of NYS is unlikely to be caused by arginine uptake inhibition alone.

To explore other possible targets in the parasite, we investigated the effect of NYS on additional membrane uptake processes. We measured the incorporation of a mixture of 10 amino acids (Asp, Glu, Gln, Gly, Leu, Lys, Phe, Ser, Trp, and Val) in the presence or absence of NYS. Since amino acids uptake in *T. cruzi* primarily occurs through permeases of the TcAAAP family (amino acid/auxin permeases), whose members share 75% of global sequence identity (Bouvier et al., 2004), it is plausible that an arginine transport inhibitor might also affect the activity of other TcAAAP proteins. Additionally, we determined the uptake of thymidine, an unrelated metabolite transported by TcrNT2, a member of the Equilibrative Nucleoside Transporters family (Campagnaro et al., 2018). In both cases, NYS at 1 μM produced a similar inhibitory effect to that observed for arginine transport. Inhibition levels were 56.0% ± 4.1 for arginine, 56.0% ± 14.5 for the amino acids mixture and 53.1% ± 8.12 for thymidine (Figure 1C). These results indicate that NYS does not act exclusively on arginine transport but also inhibits other membrane uptake processes.

**Figure 2:**
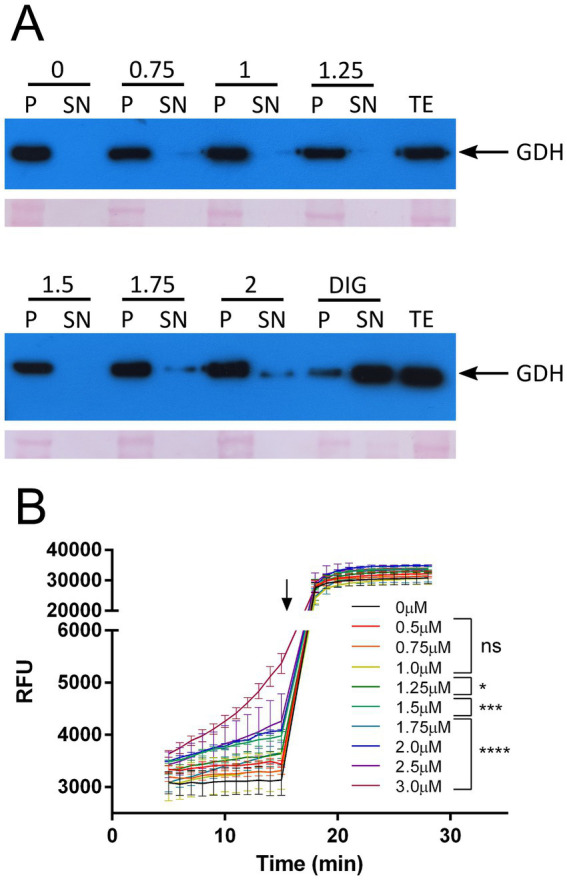
Membrane disruption by NYS. **(A)** Membrane stability was studied incubating 10^8^ parasites with increasing concentrations of NYS (from 0 to 2 μM) or digitonin as positive control (0.3 mg/mL). Supernatant and pellet fractions were analyzed by Western blot using antibodies anti-*T. cruzi* glutamate dehydrogenase (GDH) which localizes in the cytosol (Cazzulo, 1994). Ponceau red stained membranes are also shown in the figure. **(B)** Membrane integrity was quantified using EtBr in presence of NYS (between 0.5 and 3.0 μM). Arrow indicates the time at which Triton X-100 was added. Slopes were calculated for each concentration between 5 and 15 min and compared to the control condition (NYS 0 μM). NYS, nystatin; DIG, digitonin; P, pellet fraction; SN, supernatant fraction; TE, total extract; EtBr, ethidium bromide; RFU, relative fluorescence units; ns, not significant. ^*^p-value >0.05, ^***^p-value >0.001, and ^****^p-value >0.0001.

The antifungal mechanism of action of NYS has been linked to membrane sterols binding, mainly ergosterol, and pore formation (Kristanc et al., 2019). In order to investigate whether similar mechanisms could be involved in *T. cruzi*, we studied the membrane stability of the parasites through a permeabilization assay with increasing concentrations of NYS (Figure 2A). The non-ionic detergent digitonin was used as positive control and the cytosolic protein glutamate dehydrogenase (GDH) (Cazzulo, 1994) served as a marker for membrane stability. Membrane permeabilization occurred and depended on NYS concentration, since GDH was only detected on supernatant fractions over 1.75 μM NYS.

In order to quantify the membrane disruption caused by NYS, we performed an assay of ethidium bromide (EtBr) exclusion (Figure 2B). EtBr presents a fluorescence enhancement upon binding DNA, thus a compromise in membrane integrity would result in augmented fluorescence. The results confirmed that the plasma membrane is affected over 1.25 μM NYS since no significant increase in EtBr fluorescence could be observed with NYS 0.5, 0.75 and 1.0 μM (Figure 2B).

Altogether the results indicate that the mechanism of action of NYS in *T. cruzi* is through disruption of plasma membrane and at concentrations below 1.25 μM the interaction of NYS with the membrane might be enough to affect membrane transport processes.

### 3.2 Effect of NYS on trypomastigotes and the intracellular life cycle progression

**Figure 3:**
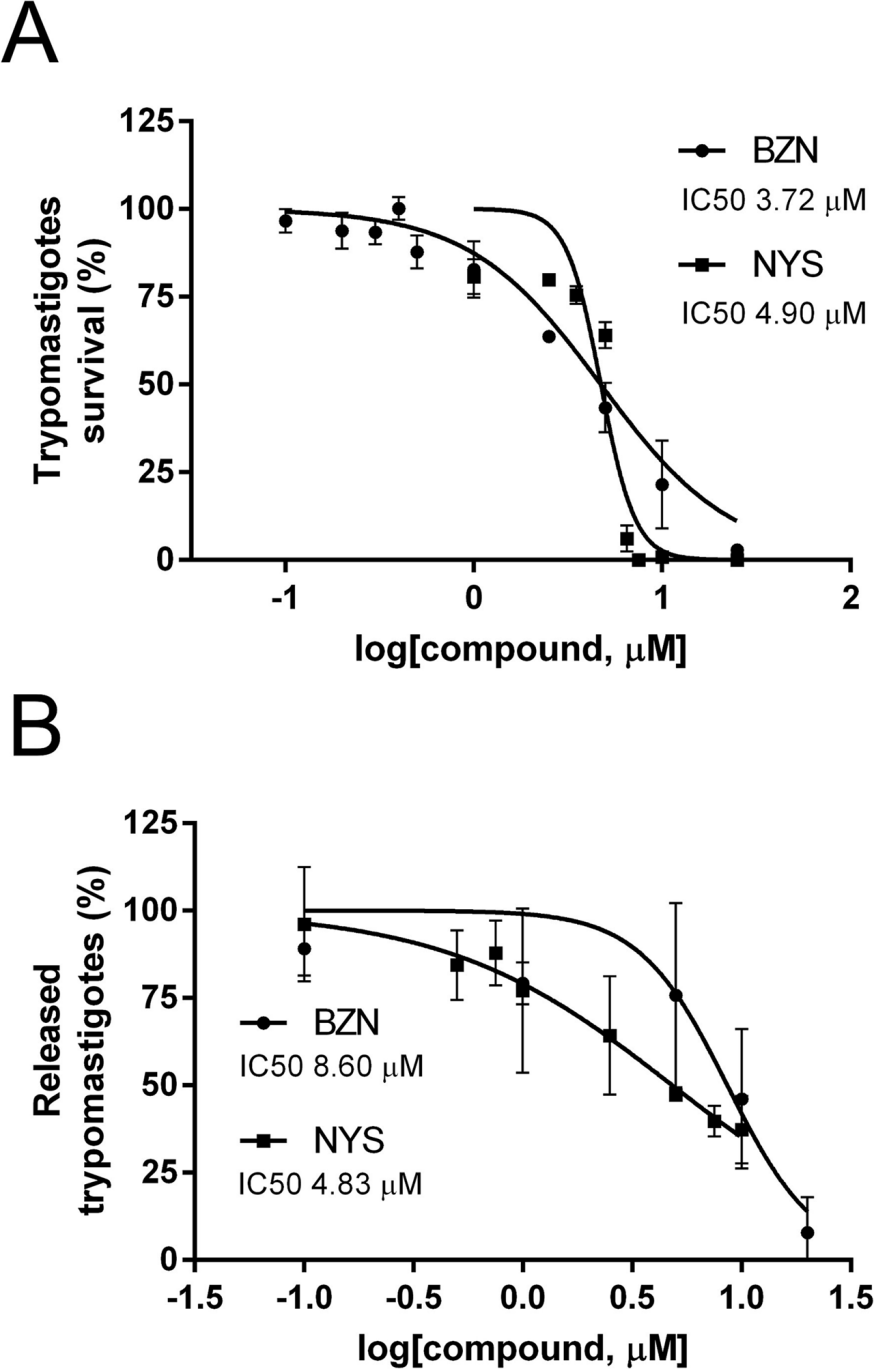
Effect of NYS on trypomastigotes and the intracellular life cycle. **(A)** Trypomastigotes were incubated for 24 h with increasing concentrations of NYS (■), or BZN (●), both from 0 to 25 μM. **(B)** Progression of intracellular life cycle was studied using increasing concentrations of the drugs in Vero cells already infected with trypomastigotes for 24 h. NYS (■) was evaluated from 0 to 10 μM and BZN (●) from 0 to 20 μM. Released trypomastigotes were counted at day 9 post-infection. Survival rate of Vero cells at NYS 5 μM was 90.32% ± 12.65. Symbols represent mean values and error bars correspond to standard deviations (*n* = 3). Concentrations of drug inhibiting the 50% of trypomastigote survival or release (IC50) were calculated using the non-linear regression of dose-response logistic functions with GraphPad Prism software. NYS, nystatin; BZN, benznidazole.

Next, the effect of NYS was evaluated on trypomastigotes, the infective and non-replicative stage of *T. cruzi* in mammalian cells (Figure 3A). The IC50s for survival of trypomastigotes derived from infected Vero cells were similar for NYS and BZN, with values of 4.90 μM (95% CI: 4.46–5.38) and 3.72 μM (95% CI: 3.16–4.38), respectively (*p*-value = 0.08).

In order to calculate the selectivity index (SI), cytotoxicity was assessed in three different mammalian cell lines: HEK293 (human), 3T3-L1 (mouse) and Vero (monkey). The SI, defined as the ratio between cytotoxicity and trypanocidal activity, reflects the drug’s selectivity for parasites over host cells. NYS presented higher selectivity towards *T. cruzi* than for the three cell lines (Table 1).

**Table 1:** NYS cytotoxicity in different mammalian cell lines.

Cell line	IC50 (95% CI)	Selectivity index
Vero	34.3 μM (17.6–66.7)	7.0
HEK293	33.4 μM (27.8–40.2)	6.8
3T3-L1	65.5 μM (40.3–106.2)	13.4

NYS effect was evaluated on three mammalian cell lines. Cell viability was assessed using gentian violet and concentrations of drug inhibiting the 50% of cell viability (IC50) were calculated using the non-linear regression of dose-response logistic functions with GraphPad Prism software. Selectivity index was calculated as the ratio between the IC50 obtained in mammalian cells and in trypomastigotes (IC50_trypomastigotes_ = 4.90 μM). NYS, nystatin; 95% CI, 95% confidence interval.

Once the trypomastigotes have entered the host cell, the progression of the intracellular life cycle includes differentiation to amastigotes, replication of these forms, differentiation back to trypomastigotes and finally the cell burst with the release of the accumulated trypomastigotes to the medium. Vero cells were infected and then the cells were exposed to increasing concentrations of the drug. After 24 h treatment, the drug was washed out and, at day 9 post-infection, released trypomastigotes were quantified as a measure of the compound’s impact on the intracellular cycle. The effect of BZN was also assessed, and NYS proved to be more effective in reducing the trypomastigote burst, with IC50 values of 4.83 μM (95% CI 3.50–6.65) for NYS and 8.60 μM (95% CI 6.23–11.87) for BZN (*p*-value = 0.03) (Figure 3B). These results indicate that NYS affects the progression of the intracellular life cycle as the drug was added after the initial host cell infection.

### 3.3 Combined treatment of NYS and BZN

The combined effect of NYS and BZN on trypomastigotes was explored, with drug interactions classified according to the combination index (CI). The CI is recognized as the standard measure of combination effect where CI < 1 means synergism, CI = 1 indicates additive effect, and CI > 1 is interpreted as antagonism. To preliminary analyze the interaction on isolated trypomastigotes, we selected sub-IC50 concentrations of NYS and BZN and constructed a 3 × 3 combination matrix using 0.1, 0.5 and 1.0 μM of each drug (Table 2). All treatments presented a synergistic effect, with CI values ranging from 0.07 to 0.62, suggesting that the two drugs were more effective in combination than individually. In addition, the dose-reduction index (DRI), which refers to the fold reduction in individual drug dosage achievable through combination for a given effect, was greater than 1 for all tested combinations of NYS and BZN on trypomastigotes. The combinations of 0.1 μM BZN with 0.1 μM and 0.5 μM NYS presented the best CI and DRI values and therefore were selected for evaluation during the progression of the intracellular life cycle on infected cells (Table 3). Both combinations presented synergistic effect with CI values of 0.26 and 0.51 and DRI values for BZN of 17.76 and 41.43. The survival rate of Vero cells with these combinations was 94.26% ± 11.61 and 89.50 ± 6.55, for the combination of 0.1 μM BZN with 0.1 μM and 0.5 μM NYS, respectively. Altogether, these results suggest that the combination have potential to allow dosage reduction and thus reduce adverse effects while keeping drug efficacy.

**Table 2:** Combined treatment of NYS and BZN on isolated tryposmatigotes.

		Nystatin		
		0.1 μM	0.5 μM	1.0 μM
Benznidazole	0.1 μM	CI = 0.06 ± 0.00	CI = 0.33 ± 0.01	CI = 0.59 ± 0.14
		Fa = 0.49 ± 0.01	Fa = 0.36 ± 0.03	Fa = 0.39 ± 0.05
		DRI_BZN_ = 43.19 ± 1.95	DRI_BZN_ = 25.24 ± 2.15	DRI_BZN_ = 27.26 ± 6.33
		DRI_NYS_ = 27.54 ± 1.68	DRI_NYS_ = 3.45 ± 0.12	DRI_NYS_ = 1.88 ± 0.47
	0.5 μM	CI = 0.35 ± 0.03	CI = 0.60 ± 0.13	CI = 0.54 ± 0.10
		Fa = 0.29 ± 0.04	Fa = 0.32 ± 0.03	Fa = 0.47 ± 0.05
		DRI_BZN_ = 3.64 ± 0.31	DRI_BZN_ = 4.09 ± 0.93	DRI_BZN_ = 7.65 ± 1.01
		DRI_NYS_ = 12.99 ± 0.91	DRI_NYS_ = 2.93 ± 0.62	DRI_NYS_ = 2.52 ± 0.50
	1.0 μM	CI = 0.33 ± 0.10	CI = 0.65 ± 0.16	CI = 0.60 ± 0.01
		Fa = 0.44 ± 0.06	Fa = 0.39 ± 0.05	Fa = 0.49 ± 0.01
		DRI_BZN_ = 3.65 ± 1.13	DRI_BZN_ = 2.73 ± 0.63	DRI_BZN_ = 4.32 ± 0.19
		DRI_NYS_ = 23.19 ± 4.48	DRI_NYS_ = 3.77 ± 0.93	DRI_NYS_ = 2.75 ± 0.17

Trypomastigotes were incubated with different concentrations of NYS-BZN for 24 h and the effect on trypomastigote survival was recorded. The combination indexes (CI) were calculated using CompuSyn software (CI < 1 synergism, CI = 1 additive effect, and CI > 1 antagonism). BZN, benznidazole; NYS, nystatin; DRI_BZN_, dose reduction index for BZN; DRI_NYS_, dose reduction index for NYS; Fa, fraction affected by the drugs (0 = no effect, 1 = total effect). DRI >1 indicates favorable dose-reduction. CI, Fa and DRI are presented with their respective ± standard error.

**Table 3:** Combined treatment of NYS and BZN on infected Vero cells.

		Nystatin	
		0.1 μM	0.5 μM
Benznidazole	0.1 μM	CI = 0.26 ± 0.02	CI = 0.51 ± 0.03
		Fa = 0.31 ± 0.02	Fa = 0.47 ± 0.02
		DRI_BZN_ = 17.76 ± 1.94	DRI_BZN_ = 41.43 ± 3.90
		DRI_NYS_ = 4.87 ± 0.48	DRI_NYS_ = 2.09 ± 0.18

Cells infected with trypomastigotes were incubated with different concentrations of NYS-BZN for 24 h and the effect on trypomastigote release was assessed on day 9 post infection. The combination indexes (CI) were calculated using CompuSyn software (CI < 1 synergism, CI = 1 additive effect, and CI > 1 antagonism). BZN, benznidazole; NYS, nystatin; DRI_BZN_, dose reduction index for BZN; DRI_NYS_, dose reduction index for NYS; Fa, fraction affected by the drugs (0 = no effect, 1 = total effect). DRI >1 indicates favorable dose-reduction. CI, Fa and DRI are presented with their respective ± standard error.

## 4 Discussion

Although Chagas disease was discovered more than a century ago, there is still no effective cure for the chronic stage, where most patients are diagnosed. While posaconazole, fexinidazole and the compound E1224 showed promising results in culture and animal models, unfortunately they failed to cure Chagas in patients (Molina et al., 2014; Morillo et al., 2017; Torrico et al., 2023; Torrico et al., 2018; Pinazo et al., 2024). The development of new therapies to replace or complement conventional treatments remains an urgent need. In the search for these new therapeutic alternatives, drug repurposing and multidrug therapy have emerged as attractive strategies.

Drug repurposing, also known as drug repositioning, involves using an approved drug to treat a different pathology. This approach allows money and time savings because toxicity and pharmacokinetic profiles, among other features, are well-known and available (Ashburn and Thor, 2004). For example, miltefosine and eflornithine, initially discovered as cancer therapies are currently used to treat the trypanosomatid-caused neglected diseases leishmaniasis and human african trypanosomiasis (HAT), respectively (Andrews et al., 2014). Similarly, amphotericin B and paromomycin, which were developed originally to treat invasive mycosis and intestinal infections, are now used also for leishmaniasis (Andrews et al., 2014). Regarding Chagas disease, numerous medications have been studied to be repurposed as trypanocidal agents, including anticancer and antifungal compounds, and are reviewed in Porta et al. (2023). Targeting the TcAAAP family of amino acid and polyamines transporters in *T. cruzi*, some medicines have also been identified as trypanocidal compounds with potential to be repurposed. Isotretinoin, a drug used for severe acne, inhibits amino acids and polyamines uptake mediated by TcAAAP permeases and exhibits trypanocidal activity (Reigada et al., 2017). In a murine model of chronic Chagas disease, isotretinoin performed better than BZN at reducing parasitemia and anti-*T. cruzi* antibodies levels as well as preventing tissue damage (Rial et al., 2023). Other drugs evaluated as candidates for drug repurposing include the antihistamines loratadine and cyproheptadine, as well as the antibiotic clofazimine, all of which have been identified as proline uptake inhibitors with trypanocidal activity (Sayé et al., 2020). Recently, liposomal formulations of loratadine and isotretinoin have also demonstrated efficacy against the parasite (Reigada et al., 2024).

Through a bibliographical search, NYS stood out for inhibiting arginine uptake in *S. cerevisiae* and also for being a medication already approved by health control agencies. Since arginine availability affects the viability of *T. cruzi* (Miranda et al., 2012), compounds that alter the intracellular pool of this amino acid could exhibit trypanocidal activity. In this work, we investigated the potential of NYS to be repurposed as trypanocidal agent against *T. cruzi*. Previous reports have explored NYS as a possible repurposed drug for other pathologies such as migraine and viral infections with HIV-1 and SARS-CoV-2 (Selvam et al., 1993; Girotra et al., 2017; Virág, 2021). Furthermore, NYS presents leishmanicidal activity against promastigotes and amastigotes from several *Leishmania* species and also inhibits parasite entry into macrophages (Ali et al., 1997; Tewary et al., 2006; Yamamoto et al., 2018).

In this work, NYS presented trypanocidal effect and inhibited membrane uptake processes prior disruption of the plasma membrane. NYS affected membrane integrity in *T. cruzi* epimastigotes in a dose-dependent manner, as evidenced through the permeabilization and EtBr exclusion assays. These results are consistent with the primary mechanism of action of NYS in yeasts that involves sterol binding and pore formation (Kristanc et al., 2019). In *T. cruzi*, pores would begin to form after incubation with 1.25 μM NYS, allowing EtBr entry and enhanced fluorescence. At 1.75 μM NYS larger pores would be occurring that facilitate the release of proteins like GDH. In addition, NYS altered arginine transport in the parasite as well as the uptake of other amino acids and an unrelated metabolite such as thymidine prior to membrane destabilization, suggesting the non-specificity of NYS over these permeases and that NYS interaction with the membrane might be enough to alter membrane processes.

While the effect of NYS in epimastigotes was stronger than the effect of BZN, in trypomastigotes both drugs presented similar trypanocidal activity. This difference could be related to the membrane composition in these parasite stages and to the different affinity of NYS to cholesterol and ergosterol. Indeed, the plasma membrane of trypomastigotes is thinner than the membrane of epimastigotes suggesting different lipid and protein content (De Souza et al., 1978). For example, ergosterol is the major neutral lipid in the epimastigote forms (Franco Da Silveira and Colli, 1981), while in amastigotes, endogenous sterols are scarce and cholesterol is predominant and it is obtained from the host cell (Liendo et al., 1999). NYS has more affinity for ergosterol than cholesterol, and accordingly, we observed the higher effect of NYS in epimastigotes.

Since drug response can vary between different cell lines, it is important to assess cytotoxicity in diverse models. In this work we evaluated NYS cell lines from various mammalian origins, including human, mouse and monkey cells. Cytotoxicity was cell line-dependent, with Vero cells being more sensitive to NYS than HEK293 and 3T3-L1 cells. Nevertheless, we studied the progression of the intracellular life cycle in Vero cells because it is our routine *in vitro* infection model.

Combined therapy can offer advantages, such as delayed drug resistance emergence, reduced therapeutic dose and shorter treatment duration, ultimately improving efficacy and minimizing side effects. Although multidrug treatment may seem complex to implement, this approach has been successfully applied to treat neglected diseases, like leprosy (World Health Organization, 2004) and other trypanosomatid-caused diseases. For example, treatment regimens for leishmaniasis often include pentavalent antimonials as first-line drugs alone or in combination with other compounds such as miltefosine or amphotericin B (Berbert et al., 2018). Similarly, nifurtimox-eflornithine combined therapy, referred as NECT, is used to treat the second stage of *T. b. gambiense* HAT (Yun et al., 2010). These examples demonstrate the therapeutic potential of drug combination for neglected diseases. In this work, we explored the combined action of NYS and BZN on isolated trypomastigotes, resulting in a synergistic effect for all the assayed combinations. For example, the addition of 0.1 and 0.5 μM NYS to 0.1 μM BZN yielded the best CI and DRI values for BZN with a death effect of 0.36–0.49, where 1.0 equals maximum effect, and therefore were selected to be evaluated in the progression of the intracellular cycle. Both combinations presented synergistic effect on infected Vero cells, with CI values of 0.26 and 0.51. The combined treatment with 0.1 μM BZN and 0.5 μM NYS produced 47% death, allowing to reduce 41-folds the dose of BZN needed to individually achieve the same effect. The synergistic combinations have potential to allow reducing BZN dose which could lead to better tolerance and safer treatment while keeping drug efficacy, although further *in vivo* studies should be carried out to better understand the combined effect of these drugs.

NYS is prescribed primarily for candidal infections of the mucosa, skin, intestinal tract, and vagina. Although NYS is mainly administered topically, alternative drug delivery systems containing NYS have been explored and are reviewed in Sousa et al. (2023). Here we demonstrate that free NYS effectively kills the parasite *T. cruzi* in its epimastigote and trypomastigote forms with superior or equal efficacy to BZN. NYS alters membrane uptake processes prior to plasma membrane disruption and also affects the progression of the intracellular life cycle, diminishing the burst of trypomastigotes. Moreover, NYS exhibits synergistic effects in combination with BZN both in isolated trypomastigotes and on infected cells. We propose NYS as a promising candidate for drug repurposing to combat *T. cruzi*. However, further studies in animal models are needed to confirm NYS efficacy as an alternative therapy for Chagas disease.

## Data Availability

The raw data supporting the conclusions of this article will be made available by the authors, without undue reservation.

## Footnotes

^1^ http://combosyn.com
